# Validating a gamified size perception task for identifying cognitive profiles in children: a latent profile analysis of executive function and sensory measures

**DOI:** 10.3389/fpsyg.2026.1752788

**Published:** 2026-05-11

**Authors:** Nan Wang, Changsen Zhu, Yixuan Huang, Zhaowen Zhou, Yuchang Li, Cheng Deng, Zhuoming Chen

**Affiliations:** 1 Department of Rehabilitation Medicine, The First Affiliated Hospital of Jinan University, Guangzhou, China; 2 Department of Rehabilitation Medicine, West China Xiamen Hospital of Sichuan University, Xiamen, China; 3 Department of Children's Health Care, Zhongshan Development Zone People's Hospital, Zhongshan, China

**Keywords:** children, executive function, gamified assessment, latent profile analysis, sensory integration, size perception, transdiagnostic

## Abstract

**Introduction:**

Size perception is a fundamental visuospatial skill that supports children's daily functioning and academic learning. Traditional variable-centered approaches often overlook heterogeneity in cognitive strategies, particularly among children with neurodevelopmental conditions.

**Methods:**

A transdiagnostic sample of 652 children (541 typically developing, 58 with autism spectrum disorder, 53 with global developmental delay) completed a nine-level gamified size perception task and standardized assessments of executive function, illusion susceptibility, and sensory integration. Latent profile analysis (LPA) was applied to external cognitive and sensory variables. Confirmatory factor analysis (CFA) tested the task's factor structure. Within-profile paired t-tests (Bonferroni correction) examined differences across latent factors. Receiver operating characteristic (ROC) analysis evaluated screening accuracy.

**Results:**

LPA yielded three stable profiles: Low Cognitive-Sensory (*n* = 108), Moderate Cognitive (*n* = 373), and High Cognitive (*n* = 171). CFA identified three latent factors: Basic Visual Discrimination, Sequential Visual Working Memory, and Perceptual Conflict Control. Within-profile comparisons showed that the High Cognitive profile scored significantly higher on the two executive-demanding factors than on Basic Visual Discrimination (both *p* < 0.001), whereas no such differences were observed within the other two profiles. ROC analysis demonstrated excellent discriminative ability for identifying children in the high-risk Low Cognitive-Sensory profile (area under the curve = 0.92, sensitivity = 85.2%, specificity = 85.5%).

**Discussion:**

Different cognitive profiles employ qualitatively different strategies: the High Cognitive profile flexibly engages executive resources under high task demands, while the other profiles rely on basic perceptual processing regardless of task complexity. The gamified task is brief, engaging, and suitable for large-scale screening. These findings support a person?centered approach to assessing cognitive heterogeneity and highlight the utility of gamified assessments for early identification of children at risk for cognitive difficulties.

## 1 Introduction

Children constantly make decisions that require accurate size perception in their daily lives: selecting the larger of two objects, fitting a block into a corresponding opening, or producing handwriting that respects spatial boundaries. Size perception—the ability to accurately judge object dimensions despite variations in viewing distance, context, and retinal image size—is a fundamental visuospatial skill that supports both daily functioning and academic learning (Cantlon, 2020; Gunderson et al., 2012). It enables children to adjust their grip when handling objects, coordinate movements during play, and navigate uneven terrain. In the classroom, proficient size perception underpins legible handwriting and mathematical reasoning, including quantity comparison, graph interpretation, and the development of a robust number-space association (Kaiser et al., 2015; Gunderson et al., 2012). Conversely, difficulties in this basic perceptual ability are associated with cascading problems in adaptive behavior, academic confidence, and social participation, potentially leading to long-term learning and social challenges (Vicari et al., 2006; Ghisi et al., 2016).

Despite its clinical and educational significance, research on children's size perception has been dominated by variable-centered approaches—correlation, regression, and group-mean comparisons—that assume homogeneity within diagnostic categories. These methods are designed to reveal linear relationships among variables and characterize the “average” child in a population (Muthén, 2001). However, they carry a hidden but critical limitation: they average over latent subgroups that may employ qualitatively different cognitive strategies or exhibit distinct patterns of strengths and weaknesses (Feczko et al., 2019; Pennington, 2006). A variable-centered analysis might conclude that “working memory predicts size perception accuracy” without recognizing that this relationship holds only for a subset of children, while another subset relies primarily on sensory strategies, and yet another shows no systematic association. This problem, known as the ecological fallacy, means that models accurately describing the average child may fail to describe any individual child (Garon et al., 2008).

The limitation is particularly acute in neurodevelopmental conditions such as autism spectrum disorder (ASD) and global developmental delay (GDD), where intra- and inter-individual variability is the rule, not the exception (Happé and Frith, 2020; Lord et al., 2020). A child with ASD may show enhanced local processing but poor global integration; another with the same diagnosis may present the opposite pattern (Mottron, 2021). Children with GDD encompass a wide range of etiologies and complex, often overlapping patterns of cognitive impairment (Shevell, 2015). Group-level comparisons between ASD and typically developing (TD) children inevitably obscure such heterogeneity. Moreover, variable-centered approaches cannot identify TD children without a formal diagnosis who nonetheless exhibit specific perceptual-cognitive difficulties and could benefit from targeted support.

Executive functions are expected to relate to size perception performance for specific theoretical reasons. Working memory is required to actively maintain and compare multiple size representations across trials or within a single trial such as seriation tasks. Inhibitory control is needed to suppress misleading retinal image information when judging real-world size, particularly in size-constancy illusion tasks where the physically larger object may appear smaller on screen. Cognitive flexibility supports switching between different task rules, such as shifting from “which is bigger on the screen” to “which is bigger in real life.” Thus, individual differences in these executive components should systematically influence performance on the size perception task, especially on complex levels that impose high cognitive load.

### 1.1 A person-centered alternative

A powerful alternative is latent profile analysis (LPA), a finite mixture modeling technique that classifies individuals into homogeneous subgroups based on their response patterns across multiple continuous indicators (Berlin et al., 2014; Lubke and Muthén, 2005). Instead of asking “how do variables relate to each other?,” LPA asks: “does this heterogeneous sample consist of smaller, latent subgroups that exhibit similar performance patterns?” This person-centered approach has gained substantial traction in executive function (EF) research. Recent studies have revealed that children exhibit qualitatively distinct EF profiles rather than falling along a single continuum. For example, Brandt identified four EF profiles in a community sample of 1,657 children, which predicted later self-regulation outcomes (Brandt et al., 2024). Chaku found four meaningful EF profiles across 11,672 youth, with profile membership predicting externalizing and internalizing problems 1 year later (Chaku et al., 2022). Vaidya conducted a data-driven identification of EF subtypes across TD, ADHD, and ASD, revealing three transdiagnostic subtypes better distinguished by neurobiological engagement than by DSM diagnoses (Vaidya et al., 2020). A recent systematic review and meta-analysis (Sadozai et al., 2024) concluded that EF delay is a robust transdiagnostic feature of neurodevelopmental conditions, with a moderate effect size (Hedges's *g* = 0.56) that increases with comorbidities (Hedges's *g* = 0.72). Together, these findings demonstrate that meaningful, clinically informative cognitive profiles can be identified across diagnostic boundaries.

Building on these advances, the present study extends the person-centered approach to the domain of perceptual decision-making—specifically, size perception. To our knowledge, no study has used LPA to derive cognitive phenotypes of size perception that cut across diagnostic boundaries.

### 1.2 The need for scalable, engaging assessment tools

A further practical gap exists. Traditional EF or perceptual tests often require trained administrators, take considerable time, and may not hold the attention of young children, particularly those with attention or sensory difficulties. This limits their utility for large-scale screening in schools or community settings. Gamified assessments—computerized tasks presented as touch-screen games—offer a promising solution. They are brief, intrinsically motivating, and can be administered without specialized training (Aneni et al., 2023). However, to date, no gamified size perception task has been validated against independent cognitive and sensory measures in a transdiagnostic sample. It remains unclear whether such a task measures distinct cognitive processes (e.g., working memory load vs. conflict control) rather than merely a unidimensional difficulty gradient, and whether it can serve as an accurate first-line screener for children at risk of cognitive difficulties.

### 1.3 The present study

To address these gaps, we recruited a transdiagnostic sample of 652 children (541 TD, 58 ASD, and 53 GDD). All children completed a gamified size perception task with nine levels of increasing cognitive demand, ranging from basic perceptual comparisons to complex real-world size judgments that create conflict between retinal image size and semantic knowledge. We also collected standardized measures of EF (including working memory, inhibitory control, and cognitive flexibility), visual size constancy illusion susceptibility, and sensory integration Scale. Crucially, we applied LPA not to the task performance itself which might reflect only a difficulty gradient but to the external cognitive and sensory variables—EF scores, illusion score, and sensory integration subscales—to derive data-driven cognitive phenotypes. We then validated these phenotypes by examining their performance on the gamified size perception task. This two-step approach directly responds to the concern that task-only LPA might capture gradations of a single continuous ability rather than qualitatively distinct cognitive strategies.

We asked three research questions: (1) Do external cognitive and sensory measures yield stable, interpretable profiles in a transdiagnostic sample of children? (2) Do these profiles differ systematically on the gamified size perception task, particularly on complex levels that tap working memory and conflict control? (3) Can the gamified size perception task serve as an accurate screening tool for identifying children in the highest-risk cognitive profile?

We hypothesized that: (1) LPA would identify multiple distinct cognitive profiles that cut across diagnostic boundaries, demonstrating the value of a transdiagnostic, person-centered approach; (2) profiles with poorer EF would show disproportionately worse performance on complex task levels, supporting the role of EF as protective factors; and (3) the gamified task would demonstrate excellent screening accuracy (AUC > 0.90) for the low-performing profile, supporting its utility as a practical first-line screener.

## 2 Methods

### 2.1 Participants

The study recruited 652 children from kindergartens and special education centers in urban Zhongshan, China. The final sample consisted of 541 TD children, 58 children diagnosed with ASD, and 53 children with GDD. To be eligible for the study, all children had to meet the following criteria: (1) a mental age between 3 and 7 years; (2) normal or corrected-to-normal vision; and (3) the ability to understand the concepts of “big” and “little” and to follow simple instructions. For the clinical groups, a formal diagnosis of ASD by a qualified clinician using DSM-5 criteria or a documented delay in at least two developmental domains for GDD was required. We excluded any child with significant motor or sensory impairments that would interfere with task completion.

### 2.2 Measures and procedure

#### 2.2.1 Size perception assessment

We assessed children's size processing abilities using a gamified computerized system (Wang et al., 2025). This tool measured skills ranging from basic perceptual comparisons to complex decisions about abstract size concepts. Testing took place in a quiet room with adequate lighting to maintain optimal conditions.

**Figure 1:**
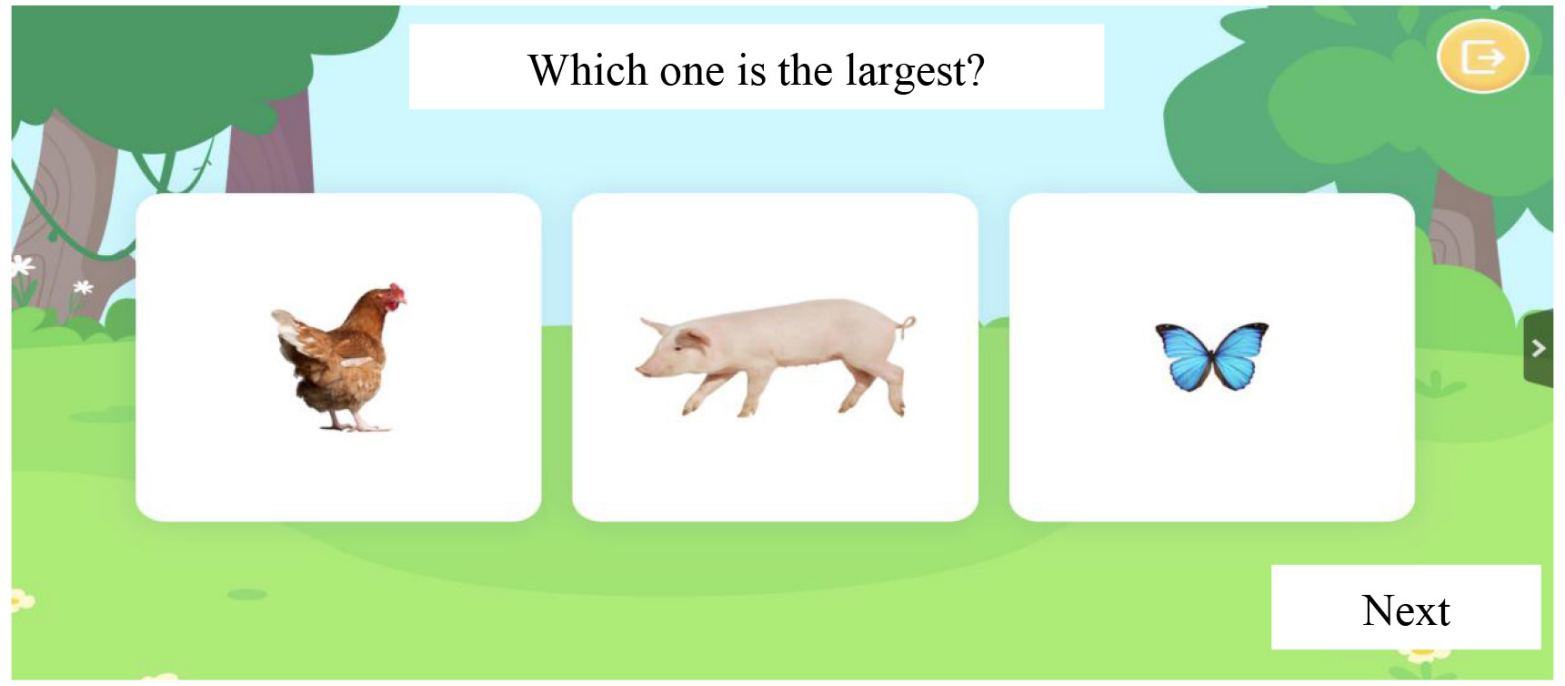
Presentation interface of size perception gamified computerized system (Take level 3 as an example).

Stimuli appeared on a 14-inch touchscreen laptop. Both the researcher and child sat on the same side of the screen during sessions. We positioned children directly facing the screen, with their eye level matching the screen's center to maintain consistent viewing geometry. All image pairs appeared symmetrically along the screen's central vertical axis (Figure 1).

The test materials featured colorful animal images familiar to children. Each animal appeared in two different poses and five sizes (260 × 260, 150 × 150, and 80 × 80 pixels). Before testing began, children completed a practice session to learn the task requirements and touchscreen operation.

The task comprised nine levels of increasing cognitive demand, each with 10 trials. Accuracy (percentage correct) was recorded for each level. The levels were:

Level 1–2: Basic perceptual comparison (two items, same or different animals). Instruction: “Which one is bigger?”

Level 3: Size Seriation—Largest (Three-items, different animals): Children identified the largest animal among three different animals of randomly assigned sizes. Instruction: “Which one is the largest?”

Level 4: Size Seriation—Medium (Three-items, different animals): Children identified the medium-sized animal among three different animals of randomly assigned sizes. Instruction: “Which one is medium-sized?”

Level 5: Complex Size Seriation (Three-items, different animals): Children identified the largest, medium, or smallest animal among three different animals, with the target dimension varying per trial. Instruction: “Which one is the largest/medium-sized/smallest?”

Level 6: Size Seriation (Five-items, different animals): Children identified the largest or smallest animal among five different animals of randomly assigned sizes. Instruction: “Which one is the largest/smallest?”

Level 7: Real-World Size Judgment (Size-matched, different animals): Children judged which of two different animals was larger in real life, while the images were presented at the same pixel size. Instruction: “Which one is bigger in real life?”

Level 8–9: Real-world size judgment with size-mismatched images, creating conflict between retinal image size and real-world knowledge. Instruction: “Which one is bigger in real life?” (Level 8) or “Which one is the largest/smallest in real life?” (Level 9).

For each subtest, both accuracy (percentage of correct responses) and average reaction time (in seconds) for correct trials were recorded as primary dependent variables.

#### 2.2.2 Executive function assessment

**Inhibitory control:** Assessed using the Head-Toes-Knees-Shoulders (HTKS) task (Fernandes et al., 2023). The task consists of 20 items scored 0~2 points each (total score 0~40), with higher scores indicating better inhibitory control (α = 0.90).

**Working memory:** Evaluated using digit span forward and backward tasks (Pineau et al., 2019). Testing discontinued after two consecutive failures at a given sequence length. Maximum scores were 16 for each task.

**Cognitive flexibility:** Measured using the Dimensional Change Card Sort Task (Mengxia, 2024), which requires children to sort cards according to changing rules. The task included 24 trials with one point for each correct sort (total score 0~24).

#### 2.2.3 Visual size constancy illusion

Participants viewed three sets of images containing figures of equal physical size presented in different spatial contexts that created illusionary size differences. Children judged whether the figures were equal in size. Scores ranged from 1 to 3 points based on the difficulty of rejecting the illusion (higher score indicates greater susceptibility to the illusion).

#### 2.2.4 Sensory integration rating scale

The Children's Sensory Integration Ability Development Rating Scale (van Sassen et al., 2025), comprising 58 items rated on a five-point frequency scale, was completed by parents. The scale yields *T*-scores (*M* = 50, *SD* = 10) for six domains: S1 (Dysfunction in Sensory Discrimination), S2 (Difficulties in Neurophysiological Inhibition), S3 (Disorder in Spatial and Form Perception), S4 (Developmental Motor Coordination Disorder), S5 (Gravitational Insecurity), and S6 (Tactile Defensiveness and Temperament Sensitivity).

### 2.3 Statistical analysis

All analyses were performed with the use of Mplus (version 8.3), R software (version 4.2.3), and Graphpad (version 10.0). Data analysis proceeded in four stages: (1) Stage 1—Latent profile analysis (LPA) on external variables (Berlin et al., 2014). We used the three executive function scores, the illusion score, and the six sensory integration subscales as indicators. All variables were standardized. Models with 1–5 profiles were estimated (mclust, VVI). The optimal number of profiles was determined by the Bayesian Information Criterion (BIC), Lo-Mendell-Rubin adjusted likelihood ratio test (LMR-LRT), bootstrap likelihood ratio test (BLRT), and entropy (Enders, 2006). For model fit evaluation, entropy values > 0.80 indicate good classification accuracy (Celeux and Soromenho, 1996). For ROC analysis, AUC values were interpreted as follows: 0.90–1.00 = excellent, 0.80–0.90 = good, and 0.70–0.80 = fair (Hosmer et al., 2013). Bootstrap resampling (500 iterations) assessed stability. The final solution had three profiles. (2) Stage 2—Comparison of profiles on the size perception task. One-way ANOVAs compared the three profiles on accuracy for each of the nine subtests. The nine ANOVAs comparing profiles on individual subtasks (acc1-acc9) were considered exploratory; to reduce Type I error, a conservative alpha of α = 0.01 was applied for these comparisons. The primary confirmatory analyses—the factor-level ANOVAs and within-profile paired *t*-tests—involved three comparisons for the factor ANOVAs and three pairwise comparisons per profile for the paired *t*-tests. Bonferroni correction was applied for the latter (α = 0.0167). Effect sizes (η^2^) were reported. *Post-hoc* comparisons (Tukey HSD) were performed for all tasks (Faul et al., 2009). (3) Stage 3—Sensitivity analysis (ANCOVA). To control for potential confounding, we repeated the comparisons with age, developmental age (DA), and diagnostic group (disease) as covariates, reporting partial η^2^ for the profile factor. (4) Stage 4—Clinical utility and screening accuracy. We examined the distribution of diagnostic groups across profiles using chi-square tests and Cramér's V. Then we performed receiver operating characteristic (ROC) analysis to evaluate the task's ability to identify children in the high-risk profile (Low Cognitive-Sensory; Robin et al., 2011). The predictor was the average accuracy on complex tasks (Levels 3–9). The area under the curve (AUC), optimal cut-off (Youden index), sensitivity, and specificity were calculated.

Additional validity evidence:

1. Confirmatory factor analysis (CFA; Brown, 2015) was performed on the nine accuracy scores to test the three-factor structure (Basic Visual Discrimination: acc1–2; Sequential Visual Working Memory: acc3,5,6,7; Perceptual Conflict Control: acc8,9). Model fit was evaluated with CFI, TLI, RMSEA, and SRMR.
2. Partial correlations between each subtest and the executive function measures were computed controlling for age, DA, and diagnostic group.
3. Missing data were negligible (< 3%). For completeness, an exploratory LPA using only the task accuracy scores was run but was unstable (bootstrap never selected the four-profile solution); its results are in Supplementary material.

All tests were two-tailed, α = 0.05.

## 3 Results

### 3.1 Three cognitive profiles based on external measures

**Figure 2:**
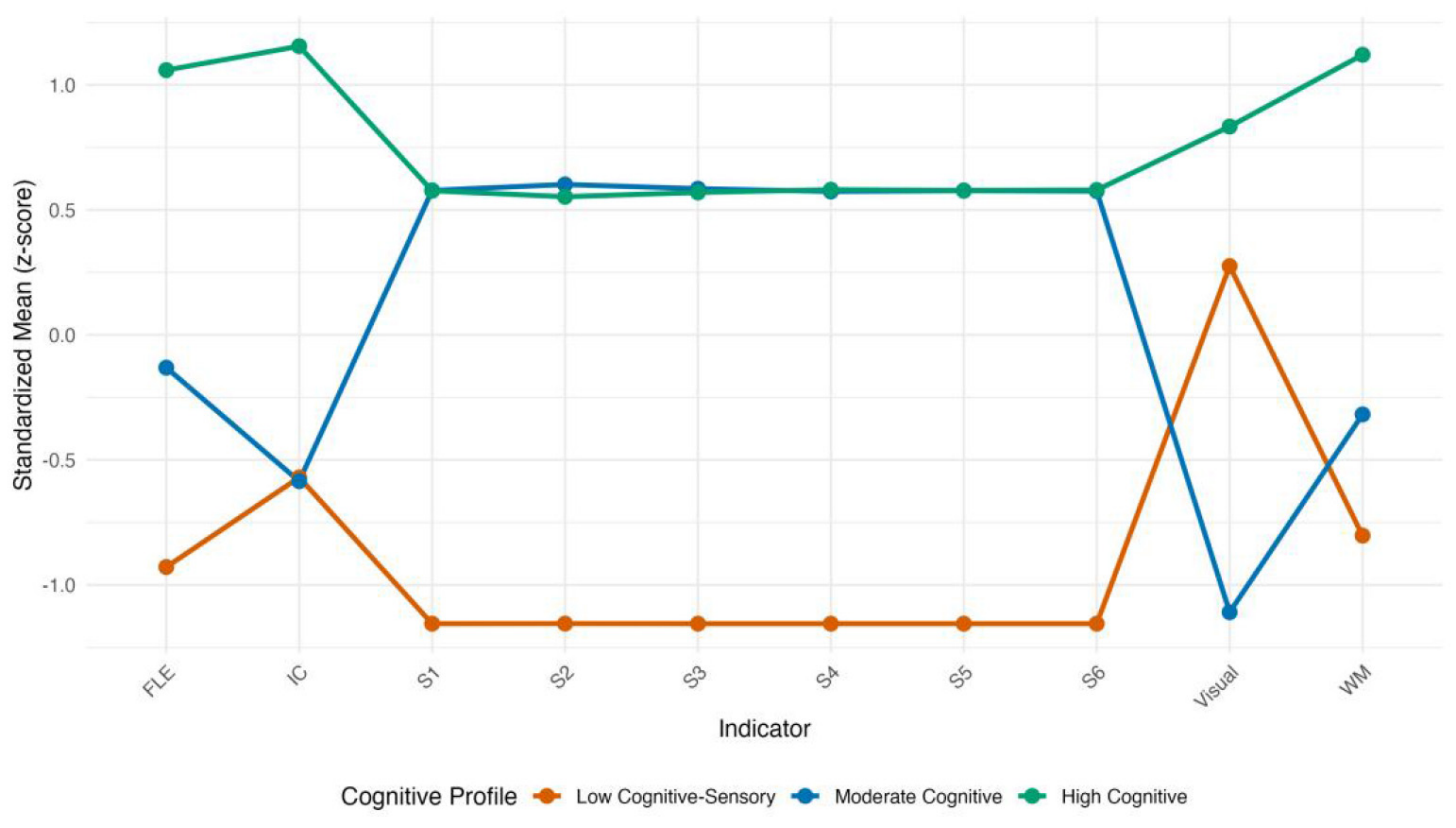
Standardized mean scores (*z*-scores) of the three cognitive profiles on executive function, illusion susceptibility, and sensory integration measures. Line plot showing standardized means (*z*-scores) of the three profiles on WM, IC, FLE, Visual, and S1–S6. Error bars represent standard errors. The dashed horizontal line at zero indicates the overall mean. Profile 1 (Low Cognitive-Sensory) is consistently below the mean, Profile 3 (High Cognitive) is above the mean on executive measures, and Profile 2 is intermediate. All variables were standardized to *z*-scores based on the full sample mean and SD.

To address whether distinct cognitive profiles exist beyond diagnostic labels, we performed LPA using executive function, illusion susceptibility, and sensory integration measures. Models with 1–5 profiles were estimated. Table 1A presents the fit indices and bootstrap stability for the 1–4-profile solutions. The three-profile solution was selected based on the lowest BIC, significant LMR-LRT (*p* < 0.001) and BLRT (*p* < 0.001), and high entropy (0.92). Bootstrap resampling (500 iterations) selected the three-profile solution in 71.2% of samples, confirming its stability. Table 1B presents the sample size and mean scores for each profile on the indicator variables, and Figure 2 shows the standardized mean scores.

**Table 1A:** Model fit indices and bootstrap stability for the three-profile LPA.

Model	AIC	BIC	aBIC	Entropy	LMR_LRT_p	BLRT_p	3-profile_percent
1	22,239.54	22,347.06	17,735.95				**71.20%**
2	19,592.83	19,758.6	17,235.56	0.95	0.01	0.01	
3	18,716.85	18,940.85	17,156.81	0.92	0.01	0.01	
4	17,411.09	17,693.33	17,106.13	0.93	0.01	0.01	

AIC, Akaike Information Criterion; BIC, Bayesian Information Criterion; aBIC, sample-adjusted BIC; LMR-LRT, Lo-Mendell-Rubin adjusted likelihood ratio test; BLRT, bootstrap likelihood ratio test.

Bootstrap resampling (500 iterations) selected the 3-profile solution in 71.2% of samples.

**Table 1B:** Characteristics of the three cognitive profiles (mean ± SD or %).

Variable	Low cognitive sensory	Moderate cognitive	High cognitive	*p*
Age (months)	86.1 ± 19.5	52.5 ± 9.9	61.0 ± 9.2	*<* 0.001
Male (%)	49.1%	44.5%	50.9%	0.341
TD (%)	0.9%	98.9%	100.0%	*<*0.001
ASD (%)	50.0%	1.1%	0.0%	*<*0.001
GDD (%)	49.1%	0.0%	0.0%	*<*0.001
DA (months)	52.7 ± 13.1	50.0 ± 10.5	61.0 ± 10.2	*<*0.001
DQ	63.1 ± 12.2	95.2 ± 9.1	100.0 ± 6.7	*<*0.001
WM	4.5 ± 2.0	5.9 ± 2.2	10.3 ± 2.8	*<*0.001
IC	7.7 ± 5.1	7.4 ± 6.3	44.4 ± 8.0	*<*0.001
FLE	5.8 ± 3.3	11.4 ± 5.5	19.8 ± 3.5	*<*0.001
Visual	2.2 ± 0.7	1.7 ± 0.8	2.4 ± 0.7	*<*0.001
S1 (Sensory discrimination)	26.3 ± 6.4	52.1 ± 1.9	52.1 ± 2.2	*<*0.001
S2 (Neurophysiological inhibition)	35.2 ± 9.2	52.1 ± 2.0	51.7 ± 1.9	*<*0.001
S3 (Spatial/form perception)	37.1 ± 10.8	52.0 ± 2.0	51.9 ± 2.0	*<*0.001
S4 (Motor coordination)	29.1 ± 6.5	52.0 ± 2.1	52.1 ± 2.1	*<*0.001
S5 (Gravitational insecurity)	19.8 ± 6.0	52.0 ± 2.0	52.0 ± 2.0	*<*0.001
S6 (Tactile defensiveness)	31.6 ± 6.1	52.0 ± 2.0	52.0 ± 2.0	*<*0.001

Data are mean ± SD or percentage.

p-values from one-way ANOVA (continuous variables) or chi-square test (categorical).

For diagnostic groups (TD, ASD, and GDD), the p-value is from the overall chi-square test [χ^2^_(4)_ = 618, p < 0.001].

DA, developmental age (months); DQ, Developmental Quotient; WM, working memory; IC, inhibitory control; FLE, cognitive flexibility; Visual, visual size constancy illusion score (range 1–3, higher scores indicate greater susceptibility).

S1–S6 are T-scores (mean 50, SD 10).

Profile 1 (Low Cognitive-Sensory, *n* = 108) was characterized by very low scores on all executive function measures and below-normal sensory integration (S1–S6 means 26–37). For instance, its inhibitory control (IC) score was only 7.7 ± 5.1, compared to 44.4 ± 8.0 in Profile 3 and 7.4 ± 6.3 in Profile 2. Profile 2 (Moderate Cognitive, *n* = 373) showed moderate executive function levels and normal sensory integration (means ~52). Its working memory (WM) and cognitive flexibility (FLE) scores were higher than those of Profile 1 but lower than those of Profile 3. Profile 3 (High Cognitive, *n* = 171) exhibited high executive function (especially inhibitory control) and normal sensory integration. For example, its WM score reached 10.3 ± 2.8, approximately twice that of Profile 2 (5.9 ± 2.2) and more than double that of Profile 1 (4.5 ± 2.0). These three profiles directly answer the first research question: there are distinct cognitive phenotypes that differ systematically in both cognitive and sensory processing.

Several patterns in Table 1B warrant explicit mention. First, Profiles 1 and 2 showed comparable levels of working memory (4.5 ± 2.0 vs. 5.9 ± 2.2) and inhibitory control (7.7 ± 5.1 vs. 7.4 ± 6.3), whereas cognitive flexibility exhibited a clear gradient across all three profiles (5.8 ± 3.3 → 11.4 ± 5.5 → 19.8 ± 3.5). This suggests that cognitive flexibility may be particularly sensitive to the cognitive demands of the size perception task. Second, the High Cognitive profile (Profile 3) had the highest illusion susceptibility score (2.4 ± 0.7), comparable to the Low Cognitive-Sensory profile (2.2 ± 0.7), yet performed best on all complex task levels. This pattern indicates that strong executive function, particularly working memory, may act as a protective factor that compensates for potentially misleading visual input (see Section 4.4). Sensitivity analysis: adjustment for developmental age. To examine whether profile membership was influenced by developmental age, we recomputed the LPA using age-residualized executive function scores (i.e., EF scores regressed on developmental age). The resulting three-profile solution showed high agreement with the original solution (adjusted Rand index = 0.904), with only minor shifts in profile sizes (Low Cognitive-Sensory: *n* = 108, Moderate Cognitive: *n* = 380, High Cognitive: *n* = 164). This indicates that the original LPA results are robust to adjustment for developmental age. Detailed results are provided in Supplementary Table S1.

### 3.2 Profile differences in size perception performance at the latent factor level

**Figure 3:**
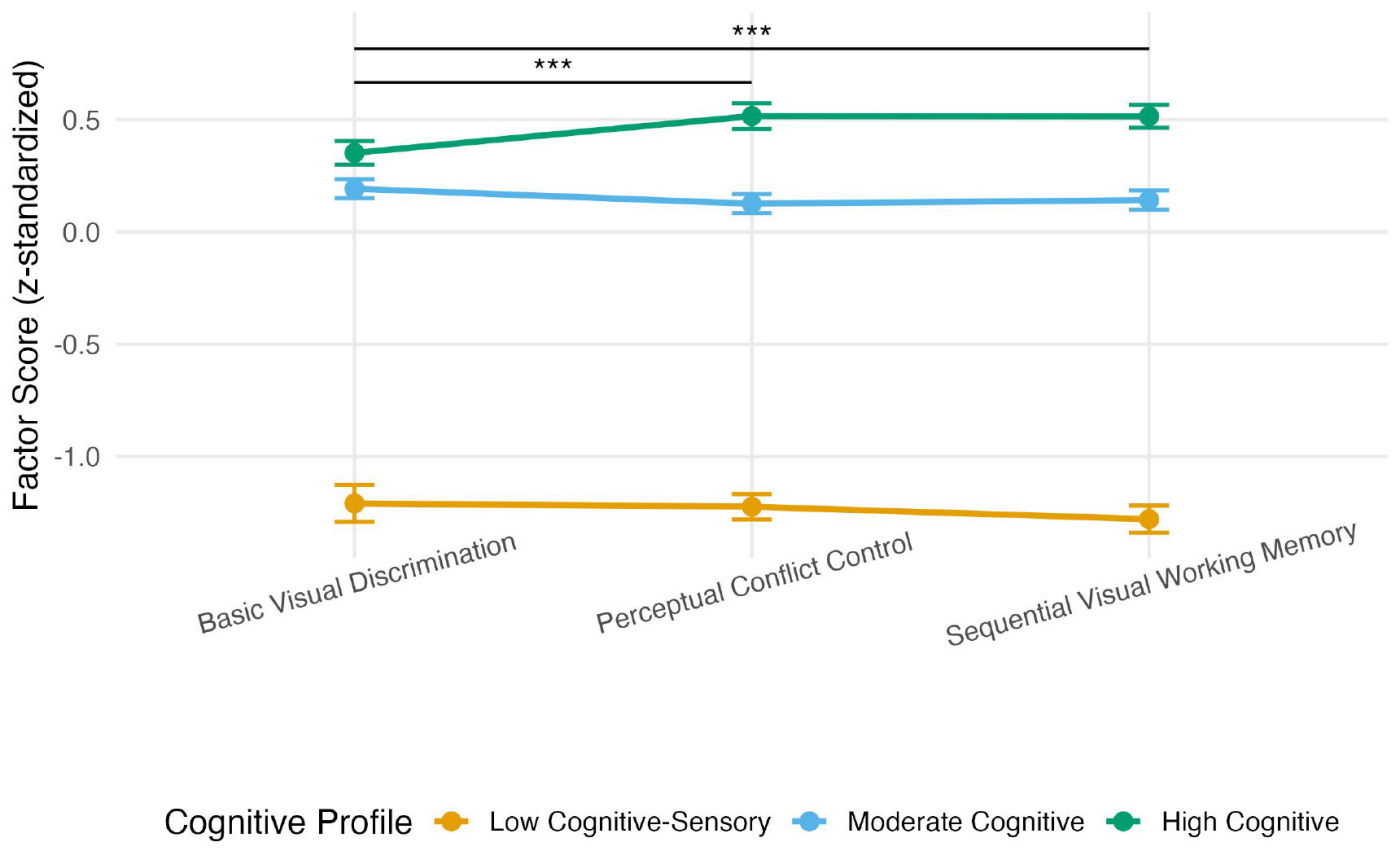
Mean factor scores (*z*-standardized) of the three cognitive profiles on the three latent factors derived from confirmatory factor analysis (CFA): Basic Visual Discrimination, Sequential Visual Working Memory, and Perceptual Conflict Control. Error bars represent standard errors. *p* < 0.001 (paired *t*-tests with Bonferroni correction, α = 0.0167) for comparisons within the High Cognitive profile: Basic Visual Discrimination vs. Sequential Visual Working Memory (upper horizontal line) and Basic Visual Discrimination vs. Perceptual Conflict Control (lower horizontal line). No significant differences were observed among the three factors within the Low Cognitive-Sensory or Moderate Cognitive profiles (all *p* > 0.017). These results indicate that the High Cognitive profile selectively engages executive resources when task demands increase, whereas the other two profiles rely on basic perceptual processing regardless of task complexity.

To reduce the number of comparisons and to test whether different cognitive profiles employ qualitatively different strategies, we compared the three profiles on the three factor scores derived from the CFA (Basic Visual Discrimination, Sequential Visual Working Memory, Perceptual Conflict Control). One-way ANOVAs revealed significant differences among the three profiles on all three factors (all *p* < 0.001), with large effect sizes for Sequential Visual Working Memory [*F*_(2, 649)_ = 196.6, η^2^ = 0.38] and Perceptual Conflict Control [*F*_(2, 649)_ = 176.4, η^2^ = 0.35], and a somewhat smaller effect for Basic Visual Discrimination [*F*_(2, 649)_ = 152.7, η^2^ = 0.32]. *Post-hoc* comparisons (Tukey HSD) confirmed that the High Cognitive profile outperformed the other two profiles on all factors, and the Moderate Cognitive profile outperformed the Low Cognitive-Sensory profile (all *p* < 0.001). Critically, to examine whether profiles differ in their pattern of performance across factors, we conducted paired *t*-tests within each profile comparing the three factors, applying Bonferroni correction (α = 0.0167). As shown in Figure 3, the High Cognitive profile scored significantly higher on the two executive-demanding factors (Sequential Visual Working Memory and Perceptual Conflict Control) than on Basic Visual Discrimination (both *p* < 0.001). In contrast, no significant differences among the three factors were observed within the Low Cognitive-Sensory or Moderate Cognitive profiles (all *p* > 0.017). This dissociation indicates that the High Cognitive profile flexibly engages executive resources when task demands increase, whereas the other profiles rely primarily on basic perceptual processing regardless of task complexity. The mean factor scores for each profile, along with the within-profile paired *t*-test results (Bonferroni correction), are presented in Table 2. For completeness, the original analyses at the level of individual subtasks (acc1-acc9) are presented in the Supplementary material (Supplementary Figure S1, Supplementary Table S2). These exploratory analyses show a broadly similar pattern across profiles, which is consistent with a difficulty gradient; however, the latent-factor analyses provide more direct evidence for strategy differences.

**Table 2:** Mean factor scores and within-profile comparisons for the three cognitive profiles.

Profile	Factor	Mean SD	Comparison	Meandiff	CI_95%	p_Bonf
Low cognitive-sensory (*n* = 108)	BasicVis	0.07 (1.02)	Bvs. S	0.07	[−0.04,0.18]	0.651
Low cognitive-sensory (*n* = 108)	SeqWM	0.00 (0.98)	Bvs.C	0.01	[−0.11,0.14]	1
Low cognitive-sensory (*n* = 108)	Conflict	0.01 (0.95)	Svs. C	−0.06	[−0.11,0.00]	0.156
Moderate cognitive (*n* = 380)	BasicVis	0.03 (0.99)	Bvs. S	0.05	[0.00, 0.11]	0.222
Moderate cognitive (*n* = 380)	SeqWM	−0.02 (0.97)	Bvs.C	0.07	[0.00, 0.13]	0.147
Moderate cognitive (*n* = 380)	Conflict	−0.01 (0.96)	Svs. C	0.02	[−0.01,0.04]	0.756
High cognitive ( *n =* 164)	BasicVis	−0.20 (0.95)	Bvs. S	−0.16	[−0.23, -0.10]	*<* **0.001**
High cognitive ( *n =* 164)	SeqWM	−0.03 (0.99)	Bvs.C	−0.16	[−0.25, -0.08]	*<* **0.001**
High cognitive ( *n* = 164)	Conflict	−0.03 (0.99)	Svs. C	0	[−0.04, 0.04]	1

Factor abbreviations: BasicVis, Basic Visual Discrimination; SeqWM, Sequential Visual Working Memory; Conflict, Perceptual Conflict Control; B, BasicVis; S, SeqWM; C, Conflict.

Data are mean (SD).

Bonferroni correction was applied for the three pairwise comparisons within each profile (*α* = 0.0167).

Bold *p*-values indicate significance after correction.

### 3.3 Sensitivity analysis: ANCOVA controlling for age, DA, and disease

To rule out the possibility that profile differences were merely due to demographic or clinical variables, we performed ANCOVAs with age, developmental age (DA), and diagnostic group (disease) as covariates. Table 3 shows that after controlling for these factors, the profile main effect remained highly significant for all nine subtests (all *p* < 0.001). Partial η^2^ for the profile factor on complex tasks ranged from 0.20 to 0.41, indicating that the profiles explain a substantial portion of variance beyond the covariates. This robustness confirms that the observed profile differences are not artifacts of age, developmental level, or diagnosis, but reflect genuine cognitive heterogeneity.

**Table 3:** ANCOVA results controlling for age, DA, and disease.

Task	Profile_F	Profile_p	Profile_eta^2^p	Age_F	Age_p	DA_F	DA_p	Disease_F	Disease_p
acc1	78	*<* 0.001	0.235	98.2	***	7.1	**	42.3	***
acc2	161.5	*<* 0.001	0.329	58.6	***	36.5	***	38.1	***
acc3	226.4	*<* 0.001	0.41	91.2	***	37.2	***	44.8	***
acc4	82.7	*<* 0.001	0.25	59.9	***	19.8	***	1.94	0.145
acc5	61.5	*<* 0.001	0.199	46.2	***	7.9	**	27.8	***
acc6	82.1	*<* 0.001	0.23	79.4	***	22.5	***	9.9	***
acc7	200.5	*<* 0.001	0.383	119.9	***	9.4	**	14.8	***
acc8	95.3	*<* 0.001	0.298	95.1	***	11.8	***	4.2	*
acc9	115.2	*<* 0.001	0.339	115.5	***	5.6	*	6.5	*

F-values are rounded to one decimal place.

***p < 0.001, **p < 0.01, and *p < 0.05.

*η*^2^*p,* partial eta-squared for the profile factor.

Degrees of freedom: profile (2,649), covariates (1,649), and disease (2,649).

### 3.4 Distribution of diagnostic groups across cognitive profiles

We then examined the clinical relevance of the profiles by analyzing the distribution of TD, ASD, and GDD children across the three profiles (Table 4). The Low Cognitive-Sensory profile consisted almost entirely of children with ASD (50.0%) and GDD (49.1%), with only 0.9% TD children. Conversely, 93.1% of children with ASD and 100% of those with GDD were classified into this profile. The Moderate and High Cognitive profiles were predominantly TD children (98.9 and 100%, respectively). The association was very strong [χ^2^_(4)_ = 618, *p* < 0.001, and Cramér's *V* = 0.69]. This result directly answers the third research question: the profiles are clinically meaningful and identify children who are most likely to need intervention, regardless of their formal diagnosis.

**Table 4:** Distribution of diagnostic groups across the three cognitive profiles.

Profile	TD_n	ASD_n	GDD_n	Row_percent	Column_percent
Low cognitive-sensory	1	54	53	0.9/50.0/49.1%	0.2/93.1/100%
Moderate cognitive	369	4	0	98.9/1.1/0%	68.2/6.9/0%
High cognitive	171	0	0	100/0/0%	31.6/0/0%

Row percentages are shown within each profile; column percentages are shown within each diagnostic group.

Chi-square test: 㱲(4) = 618, *p <* 0.001, Cramér's V = 0.69.

### 3.5 Screening accuracy of the gamified size perception task

**Figure 4:**
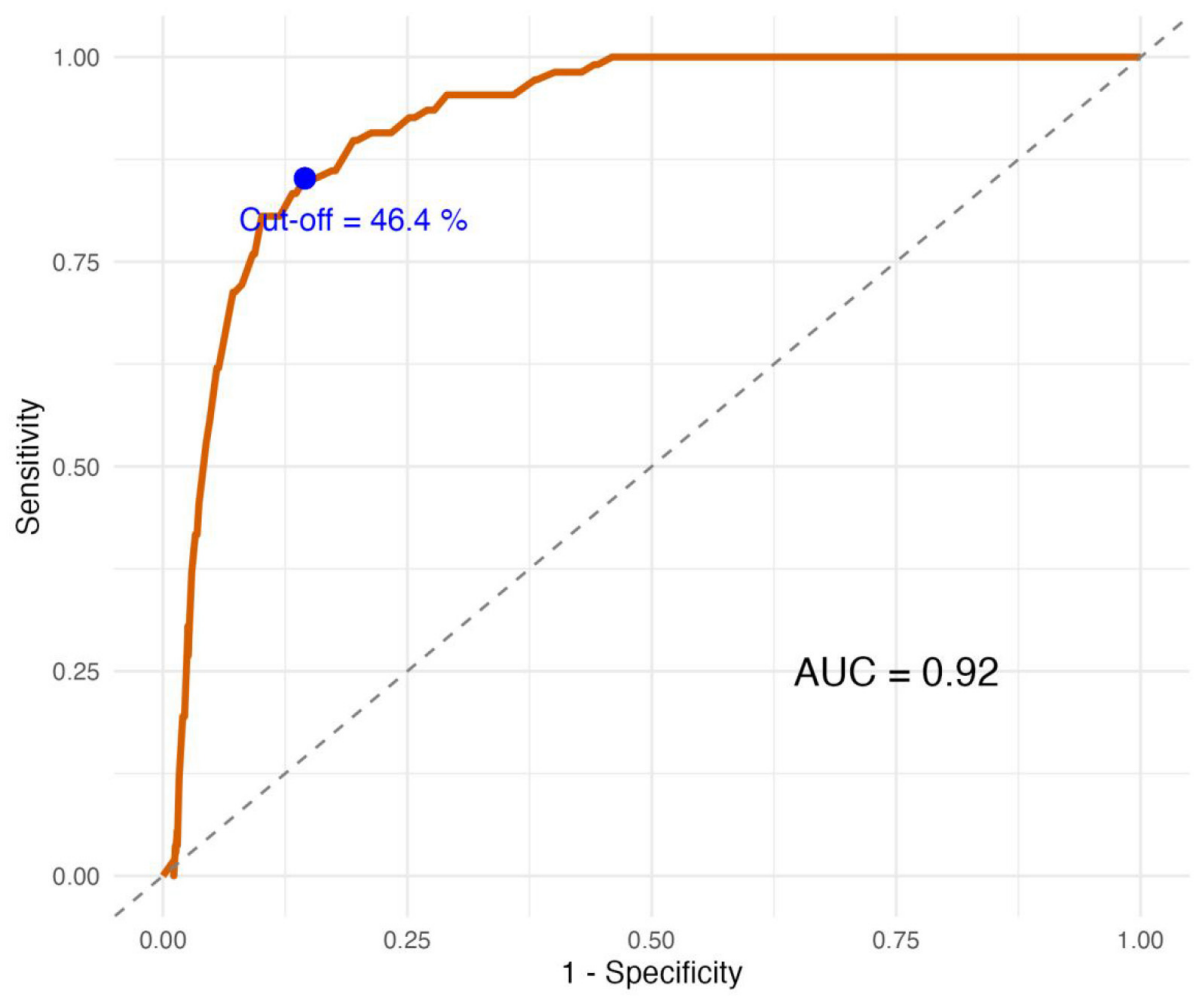
ROC curve for the gamified size perception task identifying high-risk children (Low Cognitive-Sensory profile). The area under the curve (AUC) is 0.92 (95% CI [0.897, 0.944]), indicating excellent discriminative ability. The optimal cut-off (46.4% accuracy on complex tasks) is marked by a blue point. The dashed diagonal line represents chance level.

To evaluate the task's practical utility as a screening tool, we performed ROC analysis using the average accuracy on complex tasks (Levels 3–9) to identify children in the high-risk Low Cognitive-Sensory profile. Figure 4 shows the ROC curve, with an area under the curve (AUC) of 0.92 (95% CI [0.897, 0.944]), indicating excellent discriminative ability. The optimal cut-off was 46.4% correct (Youden index), yielding a sensitivity of 85.2% and a specificity of 85.5% (Table 5). This means that the task correctly identifies over 85% of high-risk children while also correctly ruling out over 85% of low-risk children. These findings directly answer the fourth research question: the gamified size perception task can serve as a valid and accurate first-line screener for cognitive difficulties in preschool children.

**Table 5:** ROC analysis for identifying the low cognitive-sensory profile.

AUC	CI_lower	CI_upper	Cutoff	Sensitivity	Specificity	Youden
0.92	0.897	0.944	46.4	0.852	0.855	0.707

AUC, area under the curve; CI, confidence interval.

The optimal cut-off was determined by maximizing the Youden index.

### 3.6 Additional validity evidence (Supplementary material)

To further support the construct validity of the task, we conducted confirmatory factor analysis (CFA) on the nine accuracy scores. The three-factor model (Basic Visual Discrimination: acc1–2; Sequential Visual Working Memory: acc3,5,6,7; Perceptual Conflict Control: acc8,9) showed acceptable fit: χ^2^_(17)_ = 172.14, *p* < 0.001; CFI = 0.959; TLI = 0.933; RMSEA = 0.118 (90% CI [0.102, 0.134]); SRMR = 0.038. Standardized factor loadings ranged from 0.715 to 0.949 (all *p* < 0.001). Detailed results are in Supplementary Table S3. The CFA confirms that the task measures three related but distinct cognitive processes, not a unidimensional difficulty gradient.

Convergent validity was assessed by partial correlations between each subtest and the executive function measures (WM, IC, and FLE), controlling for age, DA, and diagnostic group (Supplementary Table S4). Most correlations were weak to moderate, with the strongest being acc7 with WM (*r* = 0.240, *p* = 0.028) and acc9 with IC (*r* = 0.243, *p* = 0.002). This pattern indicates that the task captures perceptual-cognitive variance that is only partially shared with traditional executive function tests, supporting its independence and added value as a screening tool.

### 3.7 Exploratory LPA on task performance alone (Supplementary material)

For completeness, we also performed an LPA using only the nine task accuracy scores (without external variables). Model fit indices (AIC, BIC, etc.) suggested a four-profile solution. However, bootstrap resampling (500 iterations) indicated that the solution was not stable enough. Therefore, we based our main analyses on the more robust three-profile solution derived from external variables. Detailed results of the exploratory LPA are presented in Supplementary Tables S5, S6 and Supplementary Figure S2.

## 4 Discussion

The present study used a transdiagnostic sample of 652 children to investigate whether cognitive profiles derived from executive function, visual illusion susceptibility, and sensory integration measures could be validated by performance on a gamified size perception task. The analysis identified three stable, clinically meaningful profiles—Low Cognitive-Sensory (*n* = 108), Moderate Cognitive (*n* = 373), and High Cognitive (*n* = 171)—that differed systematically in both cognitive-sensory characteristics and task performance. Importantly, the size perception task itself demonstrated excellent screening accuracy (AUC = 0.92) for identifying children in the high-risk profile, supporting its utility as a practical first-line screener.

### 4.1 Theoretical and clinical significance of the three profiles

The three profiles were derived from external cognitive and sensory variables rather than from task performance alone. The Low Cognitive-Sensory profile was characterized by profound deficits in all executive functions and markedly below-normal sensory integration; not surprisingly, these children performed poorly on the size perception task, especially on levels requiring working memory and conflict control. The Moderate Cognitive profile showed intermediate executive function and normal sensory integration, with intermediate task performance. The High Cognitive profile exhibited strong executive function particularly inhibitory control and normal sensory integration, performing best on all complex task levels.

The distribution of diagnostic groups across these profiles is clinically striking. Almost all children with ASD (93.1%) and GDD (100%) fell into the Low Cognitive-Sensory profile, whereas TD children were almost exclusively in the Moderate or High profiles. This finding aligns with a recent systematic review and meta-analysis (Sadozai et al., 2024), which demonstrated that executive function delays are a robust transdiagnostic feature across neurodevelopmental conditions (NDCs), with a pooled effect size of *g* = 0.56 for NDCs relative to typically developing controls, and even larger effects (*g* = 0.72) in the presence of comorbidities. The review also noted that children with ASD show particular deficits in set-switching, which echoes the extremely low cognitive flexibility scores (5.8 ± 3.3) observed in our Low Cognitive-Sensory profile. A small number of TD children (0.9%) also belonged to the low-performing profile, indicating that cognitive difficulties are not exclusive to clinical diagnoses. Conversely, a few children with ASD (1.1%) were classified as Moderate Cognitive, demonstrating that diagnostic labels alone are insufficient to predict individual cognitive profiles. This pattern supports a person-centered approach that considers cognitive and sensory profiles alongside diagnostic labels, and aligns with the Research Domain Criteria (RDoC) framework, which emphasizes dimensional constructs over categorical diagnoses (Cuthbert and Insel, 2013; Happé and Frith, 2020; Marais and Roche-Labarbe, 2025). Notably, the majority of children remained within their original diagnostic categories (Table 4), indicating that the profiles are not fully independent of diagnosis. Nevertheless, the results clearly support the utility of the task as a screening tool, as further evidenced by the ROC analysis (Table 5, AUC = 0.92).

The Low Cognitive-Sensory profile was also characterized by below-normal sensory integration across multiple domains (S1–S6 means 26–37), including tactile defensiveness and poor sensory discrimination. Such sensory regulation difficulties may compound cognitive challenges: children with this profile are likely to experience heightened sensory discomfort in testing or classroom settings, further taxing their already limited executive resources. This finding underscores the importance of holistic assessments that consider the interplay between cognitive, sensory, and emotional regulation (Green et al., 2015; Ben-Sasson et al., 2009; Klingberg et al., 2005; Mazefsky et al., 2013).

### 4.2 Differentiation of task difficulty and cognitive strategies

The present findings demonstrate that the gamified size perception task captures not merely a unidimensional difficulty gradient but rather distinct cognitive processes that are differentially engaged depending on the individual's cognitive profile. By shifting the analytical focus from nine individual subtests to three latent factors derived from confirmatory factor analysis, we were able to reduce the number of comparisons and test whether different profiles use qualitatively different strategies. The critical observation is not simply that the High Cognitive profile outperformed the other two profiles on all factors, which would only reflect differences in overall ability, but rather that the pattern of performance across factors differed between profiles. Specifically, the High Cognitive profile showed a clear dissociation: its factor scores on the two executive-demanding factors (Sequential Visual Working Memory and Perceptual Conflict Control) were significantly higher than its score on Basic Visual Discrimination, whereas the Low Cognitive-Sensory and Moderate Cognitive profiles exhibited no such within-profile differences. This pattern directly supports the hypothesis that different cognitive profiles employ different strategies. The High Cognitive profile appears able to flexibly up-regulate executive resources when task demands increase, while the other profiles rely on basic perceptual processing irrespective of task complexity.

This interpretation aligns with cognitive load theory (Sweller, 1988) and perceptual learning theory (Goldstone, 1998). Simple perceptual comparisons become automated through repeated exposure, requiring minimal executive control; consequently, all profiles performed similarly on the Basic Visual Discrimination factor. In contrast, tasks that demand active maintenance and manipulation of multiple size representations (Sequential Visual Working Memory) or suppression of misleading retinal image information (Perceptual Conflict Control) impose high cognitive load. Only individuals with sufficient executive capacity, here the High Cognitive profile, can successfully recruit working memory and inhibitory control to meet these demands. The lack of within-profile differentiation in the other two profiles suggests that their executive resources are too limited to enable strategy shifting; they remain in a default perceptual mode regardless of task difficulty.

From a predictive coding perspective (Clark, 2013; Friston, 2005), the High Cognitive profile's ability to suppress misleading visual input while maintaining accurate real-world size judgments may reflect more flexible hierarchical predictions. Their high illusion susceptibility (Table 1) indicates strong bottom-up sensory encoding, yet their superior performance on conflict tasks shows that top-down predictions can be rapidly updated when task rules change. This flexibility is absent in the Low Cognitive-Sensory profile, where sensory and executive deficits likely impair both bottom-up and top-down processes. The finding that the Moderate Cognitive profile, despite having normal sensory integration and near-typical executive scores, did not show within-profile differentiation is particularly informative. It suggests that even moderately reduced executive efficiency may prevent the strategic reallocation of cognitive resources, supporting a threshold model of executive protection.

These findings have important implications for screening and intervention. The fact that the High Cognitive profile, characterized by strong executive function and high illusion susceptibility, performs best on complex tasks suggests that executive training might enhance size perception even in individuals who are highly susceptible to visual illusions. Conversely, children in the Low Cognitive-Sensory profile may benefit from interventions that reduce cognitive load (e.g., simplifying visual displays, providing explicit strategy cues) rather than attempting to directly improve executive function. The task's excellent screening accuracy (AUC = 0.92) makes it suitable for identifying children who need such targeted support, while the factor-level analysis provides a more nuanced understanding of their cognitive strengths and weaknesses.

### 4.3 Construct independence of the size perception task

Partial correlations between subtest accuracy and executive function measures were weak to moderate (|*r*| ≤ 0.24), indicating that the task does not serve as a direct proxy for working memory or inhibitory control. Nevertheless, the three externally derived profiles differed significantly on complex task levels (η^2^ = 0.20–0.34), showing that the task is sensitive to individual differences in executive function and sensory integration without directly measuring them. This apparent paradox can be explained by cognitive load theory and perceptual learning theory.

According to cognitive load theory (Sweller, 1988), complex tasks impose high demands on executive resources; therefore, children with weaker executive function perform worse on such tasks. However, the task itself is not equivalent to an executive function test because children may use different strategies to solve the same problem. For example, in a size-seriation task, a child might rely on repeated comparisons (working-memory dependent) or on visual scanning (perceptual strategy). Children with strong executive function tend to use efficient, strategy-driven approaches, whereas those with weak executive function may depend on more basic perceptual processing. Hence, task performance reflects an indirect influence of executive function rather than a direct measure.

Perceptual learning theory (Goldstone, 1998) offers another explanation. Repeated experience can automate certain perceptual judgments, reducing the need for executive control. On the simplest tasks (Levels 1–2), all children reached ceiling, indicating that these tasks were highly automated and did not engage executive function. On complex tasks, automation was insufficient, requiring executive resources, and therefore profile differences emerged. This pattern supports the construct validity of the task.

From a neurocognitive perspective, the visual pathways involved in size perception (dorsal and ventral streams) are functionally connected to, but distinct from, the executive control network (fronto-parietal network; Grill-Spector et al., 2001; Eriksson et al., 2015). Thus, task performance can reflect the efficiency of executive function without being equivalent to it. This property of “indirect sensitivity” makes the task suitable for screening—identifying children who may need further assessment—rather than for diagnostic evaluation of specific executive deficits. The modest correlations with executive measures are actually a strength: they indicate that the task measures independent perceptual-cognitive constructs, adding value beyond existing executive function tests.

### 4.4 Executive functions as protective factors in size perception performance

The High Cognitive profile was characterized by high working memory, high inhibitory control, and the highest illusion score (most susceptible to the illusion). Despite high illusion susceptibility, this profile performed best on complex size perception tasks (Levels 3–9). This pattern supports the notion that executive functions—particularly working memory and inhibitory control—act as protective factors: strong cognitive control compensates for potential interference from misleading visual input.

From a cognitive control perspective, executive functions operate through top-down regulatory mechanisms. The prefrontal cortex generates top-down signals that bias task-relevant brain regions toward activity patterns that are optimal for the current task goal (Buschman and Miller, 2007). Inhibitory control, as a core component of executive function, is fundamental to adaptive behavior and cognition (Diamond, 2013). In complex perceptual tasks, individuals with high executive function are better able to engage fronto-parietal networks to actively maintain task goals, suppress irrelevant information, and thereby maintain accurate judgments under conflicting conditions.

Predictive coding theory offers a complementary explanation (Clark, 2013; Friston, 2005; Gabhart et al., 2025). In this framework, higher-order cortical areas generate top-down predictions that are sent to lower-level sensory regions; these predictions are compared with bottom-up sensory input, and mismatches generate prediction errors. The High Cognitive profile's tendency to judge the figures as “different” in the illusion task may reflect that their internal predictive models are more strongly weighted toward local, analytic features rather than global size-constancy inference. Nevertheless, when task instructions explicitly required real-world knowledge (Levels 8–9), this group performed optimally, demonstrating that their top-down control is highly strategically flexible.

At the neural level, the protective effect of executive functions may be related to cognitive reserve—the brain's ability to maintain performance in the face of task demands or pathology by optimizing neural network efficiency or recruiting additional brain regions (Stern et al., 2020). Children with high executive function may possess more efficient neural reserves, enabling stable performance under high cognitive load. Recent fMRI studies have further shown that the neural basis of cognitive reserve involves both enhanced efficiency of task-relevant networks (neural reserve) and compensatory recruitment of alternative regions (neural compensation; Cabeza et al., 2018).

The protective role of executive functions has transdiagnostic significance in child development. A recent systematic review and meta-analysis of children with neurodevelopmental conditions concluded that executive function deficits are a common feature across multiple neurodevelopmental disorders, whereas stronger executive functions serve as a protective factor buffering the impact of cognitive difficulties on daily functioning (Sadozai et al., 2024). Our findings are consistent with this: the Low Cognitive-Sensory profile contained a high concentration of children with ASD and GDD, whereas the High Cognitive profile contained very few clinical children, further supporting the protective role of executive functions.

Taken together, executive functions act as protective factors against perceptual challenges through top-down cognitive control, flexible modulation of predictive coding, and compensatory mechanisms of cognitive reserve. This integrated explanation not only aligns with information-processing theories that distinguish cognitive capacity from processing efficiency, but also provides a theoretical foundation for transdiagnostic, person-centered intervention strategies.

### 4.5 The gamified size perception task as a practical screening tool

The validated task offers several practical advantages over traditional executive function tests. It takes approximately 10 min, is presented as a touch-screen game, requires no trained administrator, and is highly engaging for young children. The significant separation among cognitive profiles on complex items (especially Levels 3–9) demonstrates that the task captures meaningful variance in cognitive abilities that are relevant to real-world functioning. Therefore, the task can serve as a first-line screener to identify children who may need further assessment. Its excellent screening accuracy (AUC = 0.92), high sensitivity (85.2%), and high specificity (85.5%) make it a promising tool for large-scale early identification of cognitive difficulties, with direct implications for educational and clinical practice.

### 4.6 Limitations and future directions

Several limitations should be acknowledged. First, the cross-sectional design prevents causal inferences; longitudinal studies are needed to examine whether task performance predicts later academic or functional outcomes. Second, although the secondary LPA provided robust profiles, the Low Cognitive-Sensory profile still had a relatively small sample size (*n* = 108); replication in larger, more diverse samples is warranted. Third, the original exploratory LPA based solely on task accuracy was unstable (the four-profile solution was never selected in bootstrap resampling, and the smallest profile contained only 14 children). Therefore, we based our conclusions on the more reliable three-profile solution derived from external cognitive and sensory variables. Fourth, the finding that the High Cognitive profile showed the highest illusion score requires further validation using eye-tracking or think-aloud protocols to confirm the hypothesized local processing style. Fifth, the weak to moderate correlations between task subtests and executive function measures confirm that the task measures distinct perceptual-cognitive constructs; this is a strength for screening but a limitation for diagnostic specificity. Future research should evaluate test-retest reliability, predictive validity for academic achievement, and diagnostic accuracy in clinical populations such as ASD and ADHD. Moreover, intervention studies could test whether children in the Low Cognitive-Sensory profile benefit from targeted executive function or sensory integration training, and whether improvements in task performance translate into real-world functional gains. Finally, although the present study focused on cognitive and sensory factors, emotional variables such as anxiety, stress, and emotion regulation may also influence children's size perception performance (Rabner et al., 2024). Future studies should incorporate measures of emotional functioning to provide a more holistic understanding of individual differences in perceptual decision-making.

## 5 Conclusion

This study identified three stable cognitive profiles in a large transdiagnostic sample of children based on executive function, sensory integration, and illusion susceptibility. Crucially, the High Cognitive profile showed a distinct pattern: it scored significantly higher on executive-demanding factors (working memory and conflict control) than on basic visual discrimination, whereas the other two profiles showed no such differentiation. This dissociation provides direct evidence that different cognitive profiles employ qualitatively different strategies in size perception.

The gamified size perception task demonstrated excellent screening accuracy (AUC = 0.92) for identifying children in the high-risk Low Cognitive-Sensory profile. It is brief, engaging, and requires no trained administrator, making it suitable for large-scale early screening.

These findings support a person-centered approach to cognitive assessment and highlight the utility of gamified tasks for early identification of children at risk for cognitive difficulties. Future research should adopt longitudinal designs and test profile-guided interventions.

## Data Availability

The original contributions presented in the study are included in the article/Supplementary material, further inquiries can be directed to the corresponding author.
