# Supplementary figures and images for: Validating a gamified size perception task for identifying cognitive profiles in children: a latent profile analysis of executive function and sensory measures
Source: Front Psychol. 2026 May 11;17:1752788. doi: 10.3389/fpsyg.2026.1752788 (PMC13199037; doi:10.3389/fpsyg.2026.1752788)

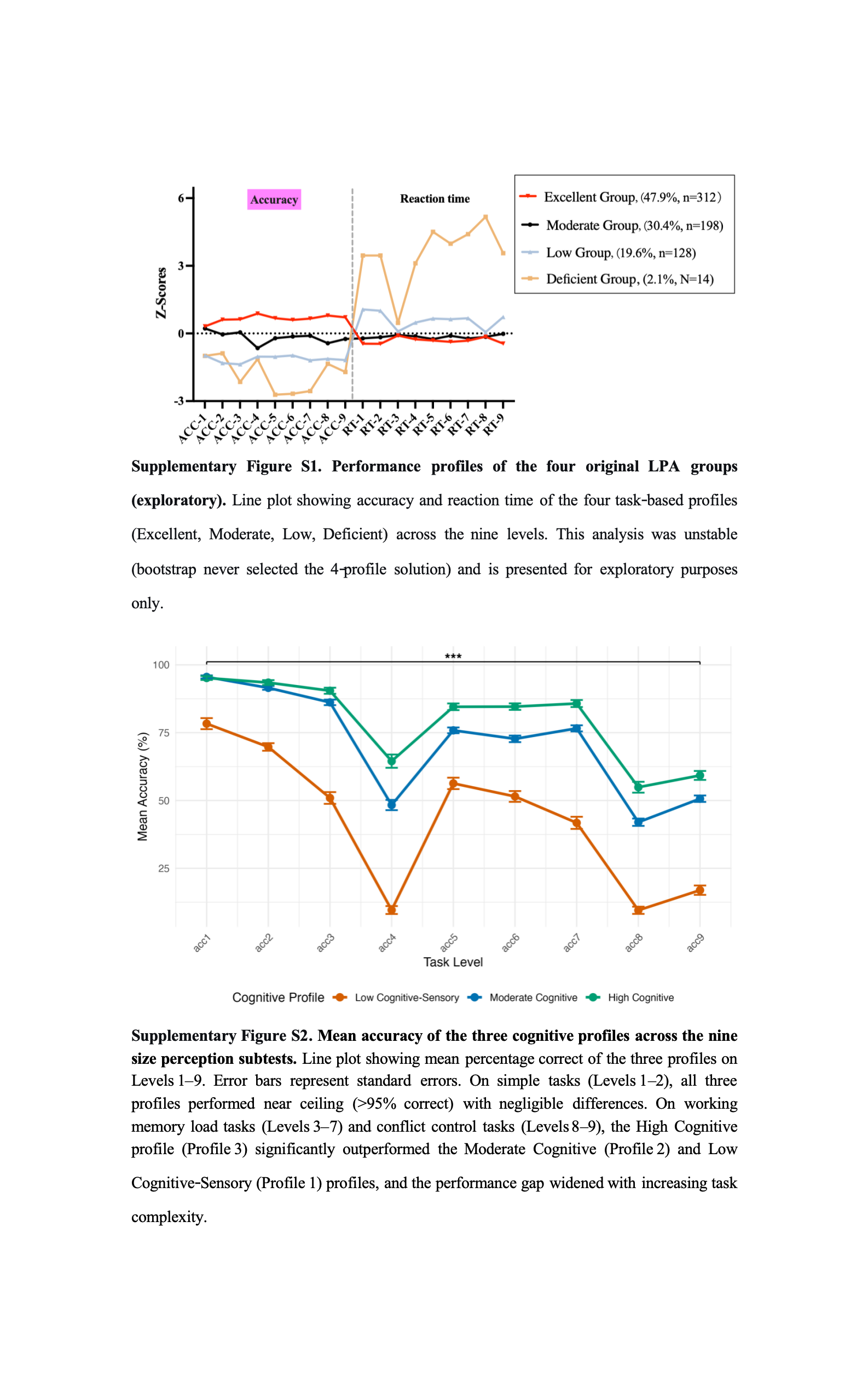

Supplement: Supplementary file 1 [file Image_1.tif]
